# What we do on social media! Social representations of schoolchildren's activities on electronic communication platforms

**DOI:** 10.1016/j.heliyon.2020.e04584

**Published:** 2020-08-20

**Authors:** Olugbenga A. Ige

**Affiliations:** University of the Free State, Bloemfontein Campus, South Africa

**Keywords:** Electronic communication platforms, Ethical issues, Facebook, Social media, School children, Cyber pornography, Cyber sexting, Cyber stalking, Abusive language, Cyber bullying, Cyber hacking, Education, Media education, Media use, Information science, Sociology

## Abstract

Researchers might be wondering whether the use of social media by students raises any ethical issues? It is my opinion that with the astronomical increase in the use of social networking websites or platforms by students, ethical issues are progressively important. The astronomical increase in the use of social networking platforms by minors raises ethical questions in school ecologies. This study investigated the ethical issues in social media usage among secondary school students in a developing context. A semi-structured questionnaire that elicited information on the school children's home background, social media operated, and mode of connection to the social media was used to collect data. The questionnaire gave the students the opportunity to write their responses to the interview questions freely. Mixed methods such as constant comparative techniques and descriptive statistical methods were used to analyze data from the one hundred and thirty school children that participated in this research. The results indicated that Facebook is the most operated social networking website by the selected schoolchildren. Most of the schoolchildren operating the popular social networking accounts signed up before the age of thirteen with the help of their biological sisters and brothers. Themes such as cyber pornography, sexting, cyber stalking, cyber bullying, cyber hacking, and abusive language emanated from the individual qualitative interviews. The concluding part of this article answers questions on the impact of new communication technologies on psycho-social adjustment of school children in developing countries.

## 1 Introduction

Why should children utilize social media in an ethical way? Scholars have concurred that this question is interesting since teachers across the world have a mission to develop children's ethical values and evaluate their ethical competences (Lilja et al., 2017:1). Yaros (2012) posited that social network sites allow personalization of users' public persona usually accompanied by communicative sharing of information with personal networks. Yaros' (2012) position implies that students can share information they would not ordinarily share in the physical space on social media. The communication possibilities of the 21^st^ century have impelled researchers in different disciplines to study the ethical dimensions in the use of social media. For instance, Denecke et al. (2015) investigated the ‘ethical issues underlying social media use in health care’ as a result of the increase in information to patients which resulted from the increased possibilities and channels of obtaining information on ailments and treatments. These ailments and treatments are usually presented on the Internet and social media platforms. Denecke et al.'s (2015) scholarly enquiry uncovered two major unethical situations. First is the unrestricted access that patients have to personal information about their physicians on the Internet. The second discovery is the access that physicians have to their patients' information on the Internet, which is not available in normal health-care settings. These ethical scenarios led the American Medical Association to recommend that health-care providers should use privacy settings to protect personal information and familiarize themselves with privacy policies of diverse social media applications. Unfortunately, these safety measures adopted by the American Medical Association becomes herculean to adopt in schools because of the dynamic nature of school ecologies, teachers' professional associations, and governing of schools.

Gelinas et al. (2017) studied ethical issues and challenges that are inherent in recruiting on the social media for research. They identified preservation of privacy and interests of other social media users, and researcher's privacy as the most essential considerations. However, this article examined ethical issues in the use of social media among secondary school students and presented a set of recommendations for teachers and researchers. These recommendations have strong implications for teaching and learning in secondary schools especially in developing nations where the Internet is still emerging.

This introductory section presents a short summary of the current literature by documenting ethical dimensions in using the social media in various disciplines and presents the research questions of this study. In the second section, the routine activity and space transition theoretical lenses were adopted to explain the ethical and unethical dimensions of social media use by secondary school students. The space transition theory is applicable to this research because it provides fundamental information on how students project their conforming and non-conforming behaviourial dispositions to other people on the social media. The routine activity theory complements space transition theory by affirming that the presence or absence of a capable guardian could enable schoolchildren use the social media appropriately or inappropriately. The third section deals with the methodology adopted for collecting data from the students that participated in the study. This section explains the preparation of research instruments, procedures used to collect data from the participants, and how the data were coded and analysed. The fourth section answers the research questions by highlighting the social media platforms usually visited or used by the participants, and the frequency of such visits. Interesting information pertaining to parents' and teachers' stances on the participants' use of social media is presented. Consequently, this article has three crucial aims: to investigate/highlight the social media commonly used by secondary school students in Nigeria; to identify the ethical and unethical activities carried out by students using the social media; and to suggest value transmission strategies that teachers can adopt to correct students' unethical behaviours on the social media.

### 1.1 Research questions

The following research questions were raised to explore the social behaviour of school children on electronic communication platforms such as 2go, Facebook, Twitter, Whatsapp, and most especially Facebook:

• What are the popular social networking websites used to communicate by secondary school students?
• What is the age range of students using the social media?
• What is the duration of the daily online engagements made by secondary school students on the social media?
• How did the selected students sign up their social media accounts?
• How do the secondary school students navigate the popular social networking websites?
• How do secondary students use social media?

With the astronomical increase in the use of social networking websites or platforms by students, ethical issues are progressively important. Deigh (2010) observed that ethics sprang from simple questions on why honest actions are right, and dishonest actions are wrong. It comprises questions that naturally arise in societies with diverse cultures and technologies. The observation of Deigh (2010) suggest that ethical issues are common in societies where children regularly use the social media. Faced with the realities of the information age, scholars have attempted to provide answers to ethical questions arising from the activities of children on the social media. For instance, Edelsohn (2012) drew attention to the fact that professionals such as clinical practitioners, educational researchers, and child and adolescent psychiatrists in public and private sectors are daily confronted with ethical issues. Maxim (2014) asserted that ethics is a methodological reflection on the principles, moral values, real life and spiritual practice of individuals and society in connection to good and evil. Taking Maxim's definition as a point of departure, this article is an assessment of the positive and negative activities of secondary school students on the social media It also tries to evaluate the level of students' awareness of their negative activities on the social media. These evaluations will assist in answering the following questions that may arise in a scenario where, for instance, James posted words that are offensive on Ade's Facebook page. What should Ade do? Should he reply to James' offensive posts? Or report James to their class teacher? Is it good for Ade to reply to James' offensive posts? Or report James to the teacher? If Ade reports James to the class teacher, is it appropriate for the teacher to resolve disagreements that arose between Ade and James on a social media website? Answers to these questions will reveal to teachers how students can perpetrate acts on the social media that are prohibited by law in the physical space and get away without being punished. They will also bring to the fore the need for teachers to teach cyber etiquette to students as it is observed that the steps a student should take when confronted with adverse situations on social media depend on whether students have received prior instructions on handling such a situation.

## 2 Theoretical framework

### 2.1 Space transition theory and students' social media usage

Space transition theory proposes that humans behave differently when they transit from one space to the other. Danquah and Longe (2011) noted that this theory was propounded by Jaishankar (2008) due to the inadequacies of conventional theories to explain the causation of crimes in the cyberspace. The theory proposed that:

1. Persons with repressed criminal behaviour in the physical space have a propensity to commit crimes in cyberspace, which, otherwise they would not commit in physical space, due to their status and position.
2. Identity flexibility, dissociate anonymity and lack of deterrence factor in the cyberspace provide offenders the choice to commit cybercrime.
3. Criminal behaviour of offenders in cyberspace is likely to be imported to physical space which, in physical space may be exported to cyberspace as well.
4. Intermittent ventures of offenders into the cyberspace and the dynamic spatio-temporal nature of cyberspace provide the chance to escape.
5. (a) Strangers are likely to unite in cyberspace to commit crime in the physical space.(b) Associates of physical space are likely to unite to commit crimes in cyberspace.
6. Persons from closed society are more likely to commit crimes in cyberspace than persons from open society.
7. The conflicts of Norms and Values of Physical Space with the Norms and Values of cyberspace may lead to cybercrimes” (p. 7).

This theory is applicable to students' ethical or unethical use of the social media in the following ways:

Students whose criminal tendencies were overpowered by social forces (i.e. elements of society which possess the capability of evoking cultural change or influencing people) could use social media to perpetrate actions that the society deems ‘bad’. In the same vein, social media users such as students are at liberty to use pseudo names (i.e. identity flexibility) for good or evil acts. This is described by Suler (2004, 2016) as dissociative anonymity, an anonymity that “works wonders for the disinhibition effect’ for people to say or do things in cyberspace that they would not do in the physical world (p. 324). The implication of this is that students can use the social media to perpetrate acts that they would not be confident to actualize in the physical space. It is observed that secondary school students venture randomly into cyberspace using social media and there is the possibility of escaping the punishment or penalties attached to crimes committed on this platform.

Another relevant postulate of space transition theory is the idea of strangers uniting in cyberspace to commit crime in the physical space. It is a possibility that students can unite with persons unknown to them in the physical space to bully and make derogatory comments about people in cyberspace. A good instance is Todd Loik, a 15-year-old who committed suicide in Canada on 8 September 2013 because of incessant taunts he received from persons unknown to him on Facebook (The Canadian Press, 2015). Also, in Sweden, the police opened an investigation on 12 March 2013 into the circumstances surrounding the death of a 13-year-old girl who was suspected to have committed suicide by stepping in front of a train about 15 km south of Örebro (Kumla). Before her death, the girl posted a video clip on the Internet complaining about the prolonged bullying she had endured on the internet. Evidence gathered by the police from IP addresses, film clips, and comments (i.e. “So ugly. Go kill yourself”) from the deceased social media's accounts revealed a boy aged 15 as the perpetrator (The Local, 12 March 2013). It is no news that secondary school students associate in physical space during and after school hours. With the escalation of information and communication technology devices, school children could team up to use social media for acts they would not engage in ordinarily in physical space.

The basic question underlying these explanations is ‘how should a teacher of Civics/Social Studies transmit appropriate values to school children who are active on the social media?’

### 2.2 Routine activity theory and secondary school students' social media use

The initial focus of Cohen and Felson's routine activity theory was the crime surges of the 1960s and 1970s that emanated from changes in daily societal life (Tilley et al., 2015). This theory is useful in explaining school children's ethical or unethical use of the Internet (see Ige, 2012; Ige, 2013). The universal application of this theory was emphasized by Tilley et al. (2015) that despite the period in which the routine activity theory emerged. Cohen and Felson (1979) defined a routine activity as a habitual and widespread activity that caters for basic population and individuals' needs. The elements of what Cohen and Felson (1979) call ‘direct-contact predatory violations i.e. cyber criminal acts that damage other users in cyberspace’ are:

I. Motivated offenders
II. Suitable targets
III. The absence of capable guardians against a violation (p. 589)

The postulates of this theory imply that secondary school students could be motivated to use social media for unethical engagements if a capable guardian is absent. But the question here is “who are capable guardians?” They are some of the agents of socialization, namely: family, teachers, and school. I exclude peer group, security institutions (see Gheciu, 2005) and the Internet because these are circumstantial or weak capable guardians. The presence of a capable guardian implies that secondary school students will adhere to ethical use of social media if properly guided. This paper presents accounts given by students about their activities on the social media. I subsequently analysed the ethical and unethical dimensions of accounts of students' social media engagements.

## 3 Research methodology

### 3.1 Data and methods

This paper adopted an after-the-fact research design that utilized an admixture of quantitative and qualitative approaches, using one hundred and thirty-five secondary school students purposely selected from different schools in Ogun, Ondo, and Delta states of Southern Nigeria. The students were purposely selected because their schools permitted this study, had email addresses and active social media accounts such as Facebook, 2go, WhatsApp etc., had unhindered access to the Internet in the selected locations, and were ready to participate in this study. These states were selected because of their relatively strong Internet services. The after-the-fact research design otherwise known as the ‘Ex post facto’ is appropriate for this study consequent on Salkind's (2010) assertion that investigation in this kind of research commences after the facts have occurred in a social milieu without interference from the researcher. These secondary schools were in urban centres in Southern Nigeria. A questionnaire entitled ‘Social Networking Media Usage Questionnaire (SNMUQ)’ was developed to investigate students' activities on the social media. The questionnaire was vetted by four graduate teachers in selected secondary schools for credibility. The suggestions of the teachers were used to rework the questionnaire before it was administered on thirty-six students to check the reliability of the questionnaire. The reliability coefficient of SNMUQ was computed using Cronbach Alpha to determine the internal consistency, and it yielded 0.77. The questionnaire elicited responses on the respondents' home background, the period spent on the social media daily, social media registered, mode of connection to the social media on which the students are active, awareness of their parents or guardians of their social media affiliations, etc. The students also responded freely to some semi-structured questions in the questionnaire (See Appendix).

#### 3.1.1 Data analysis

The quantitative data from the 135 students who agreed to participate were coded and analysed using the statistical package for social sciences 25.0. First, the qualitative data were analysed by the pattern of participants' response to the questions in the questionnaire (i.e. directly using the language of the respondents). The qualitative data collected from the participants were coded to produce an initial code until analysis had reached theoretical saturation in the opinion of the researcher using constant comparison technique as a means of analysis (see Glaser and Strauss, 1967).

#### 3.1.2 Ethical considerations

The management and students were engaged in discussions relating to the study. The researcher explained the mission of the study to the management and students of the selected schools, and subsequently sought their consent to administer the questionnaire on social networking media use. The researcher assured the students that the information given on their activities on different social media websites would be treated with confidentiality, and that pseudo names would be used where the names used on the social networking websites are their real names. The managements and students of the schools selected for this study approved of the collection and publishing of the data presented in this study for educational purpose.

#### 3.1.3 Ethical issues in social media use by secondary school students

This section answers the questions raised in this paper. The responses of respondents reveal the ethical and unethical ways they have utilized their spaces on social media. The findings from quantitative analyses, and themes emanating from the qualitative responses are further discussed to provide information to teachers on best practices of handling problems relating to students' use of the social media at the secondary school level.

## 4 Results

### 4.1 Respondents' demographic characteristics

The sample comprised 80 (59.3%) boys and 54 (40.0%) girls; 1 participant (0.7%) did not indicate his or her sex. The mean age of the participants was 12.64 (SD = 1.80). 6 (4.4%) of the participants were born in 2001, 5 (3.7%) in 2002, 7 (5.2%) in 2003, 19 (14.1%) in 2004, 38 (28.1%) in 2005, 22 (16.3%) in 2006, 19 (14.1%) in 2007, 12 (8.9%) in 2008, 2 (1.5%) in 2009, whilst 5 (3.7%) did not indicate when they were born. The mean year of birth was 2005.

The tribal background of the students shows that 50 (37.0%) are Igbo, 2 (1.5%) from Edo, 2 (1.5%) from Isoko, while 28 (20.7%) are Yoruba. The parental or home background of the students reveals that 101 (74.8%) live with their fathers and mothers, 7 (5.2%) live with fathers only, 13 (9.6%) live with mothers only, 6 (4.4%) live with grandmothers, 2 (1.5%) live with aunts, 3 (2.2%) live with grandfathers, whilst 3 (2.2%) did not disclose their background. 98 (72.6%) respondents indicated that they had mobile phones, while 34 (25.2%) had no access to mobile phones. 82 (60.7%) indicated they connected to the Internet using mobile phones to launch their social media accounts, while 48 did not. 63 (46.7%) had MTN Nigeria as their network service provider, 26 (19.3%) used Globacom, 21 (15.6%) used Airtel, 3 (2.2%) used Etisalat, 8 (5.9%) used both MTN Nigeria and Globacom as their network service providers, while 14 did not indicate their network service providers.

### 4.2 Research question one

#### 4.2.1 What are the popular social networking websites used to communicate by the selected secondary school students?

Table 1 shows that 74 (54.8%) of the selected students used Facebook, 17 (12.6%) were active on 2go, 16 (11.9%) used two or more social media, 9 (6.7%) used YouTube, 8 (5.9%) had no social media account, while 4 (3.0%) did not respond to the question.

**Table 1:** Social media used by students.

Media	Frequency	Percent	Valid percent	Cumulative percent
Facebook	74	54.8	56.5	56.5
Twitter	4	3.0	3.1	59.5
2go	17	12.6	13.0	72.5
Youtube	9	6.7	6.9	79.4
Whatsapp	3	2.2	2.3	81.7
Two or more	16	11.9	12.2	93.9
None	8	5.9	6.1	100.0
Not indicated	4	3.0	100.0	
Total	135	100.0		

### 4.3 Research question two

#### 4.3.1 What is the age range of students using the social media?

Table 2 shows that 2 (1.5%) of the respondents were 9 years old, 12 (8.9%) were10 years old, 19 (14.1%) were eleven years old, 33 (24.4%) were 12 years old, 31 (23.0%) were 13 years old, 15 (11.1%) were 14 years old, 7 (5.2%) were 15 years old, 5 (3.7%) were 16 years old, 6 (4.4%) were 17 years old, 5 (3.7%) did not indicate their ages.

**Table 2:** Age of students using social media.

Age	Frequency	Percent	Valid Percent	Cumulative percent
9	2	1.5	1.5	1.5
10	12	8.9	9.2	10.8
11	19	14.1	14.6	25.4
12	33	24.4	25.4	50.8
13	31	23.0	23.8	74.6
14	15	11.1	11.5	86.2
15	7	5.2	5.4	91.5
16	5	3.7	3.8	95.4
17	6	4.4	4.	100.0
Not indicated	5	3.7	100.0	
Total	135	100.0		

### 4.4 Research question three

#### 4.4.1 What is the duration of the daily online engagements by secondary school students on the social media?

Table 3 shows that 68 (50.4%) of the respondents spent 1–59 min on the social media daily. 23 (17.0%) spent 1hr daily on the social media, 11 (8.1%) claimed they used online media 2 h daily, 7 (5.2%) claimed they spent 3 h, 3 (2.2%) respondents they spent 5 h, 1 (0.7%) respondent spent 8 h online, 1 (0.7%) spent 15 h, 1 (0.7%) claimed to have spent 20 h, 9 (6.7%) did not log on daily, while 11 (8.1%) did not indicate the amount of time spent daily on the social media.

**Table 3:** Online engagements of students on the social media.

Duration (Time)	Frequency	Percent	Valid Percent	Cumulative Percent
1–59 min	68	50.4	54.8	54.8
1 h	23	17.0	18.5	73.4
2 h	11	8.1	8.9	82.3
3 h	7	5.2	5.6	87.9
None	9	6.7	7.3	95.2
5 h	3	2.2	2.4	97.6
8 h	1	0.7	0.8	98.4
15 h	1	0.7	0.8	99.2
20 h	1	0.7	0.8	100.0
Not indicated	11	8.1	100.0	
Total	135	100.0		

### 4.5 Research question four

#### 4.5.1 Who assisted the selected students to sign up on social media accounts?

Table 4 shows that 13 (9.6%) enlisted the assistance of friends to open social media accounts, 12 (8.9%) got assistance from their teachers to register on the social media, 7 (5.2%) respondents were assisted by their aunts to open social media accounts, 11 (8.1%) opened their social media accounts through the assistance of their fathers, 4 (3.0%) of the respondents stated their accounts were opened on the social media of their choice with the assistance of their mothers. 5 (3.7%) enlisted on the social media with the assistance of their uncles, a larger number 33 (24.4%) of the students responded their social media accounts was made possible with the help of their brothers, 4 (3.0%) responded that they were assisted by their sisters to open their social media accounts. 28 (20.7%) signed up on the social media though self-efforts, while 1 (0.7%) was helped by his cousin to open the social media account.

**Table 4:** Agents used to open social media accounts.

Agents	Frequency	Percent	Valid Percent	Cumulative percent
Friends	13	9.6	9.6	9.6
Teacher	12	8.9	8.9	18.5
Aunt	7	5.2	5.2	23.7
Father	11	8.1	8.1	31.9
Mother	4	3.0	3.0	34.8
Uncle	5	3.7	3.7	38.5
Brother	33	24.4	24.4	63.0
Sister	4	3.0	3.0	65.9
Self	28	20.7	20.7	86.7
Not indicated	17	12.6	12.6	99.3
Cousin	1	0.7	0.7	100.0
Total	135	100.0	100.0	

### 4.6 Research question five

#### 4.6.1 Who trained secondary school students how to navigate the popular social networking websites?

Table 5 shows that 12 (8.9%) learnt to operate the social media through informal trainings from friends, 7 (5.2%) learnt the operations of the social media from aunts, 16 (11.9%) respondents were taught to operate the social media by their fathers, 9 (6.7%) acquired the skills to use the social media from their mothers, 9 (6.7%) learnt to navigate the social media environments from uncles, 27 (20.0%) respondents became competent in the use of the social media through informal guide from their brothers. Sisters taught 8 (5.9%) respondents the techniques of operating the social media, 26 (19.3%) of the respondents learnt to use the social media through auto didactism, 19 (14.1%) did not answer the question, 1 (0.7%) respondent claimed they learnt to use the social media through the help of many people, while 1 (0.7%) became familiar with the operations of the social media through assistance from a cousin.

**Table 5:** Agents used to learn use of social media.

Agents	Frequency	Percent	Valid Percent	Cumulative Percent
Friends	12	8.9	8.9	8.9
Aunt	7	5.2	5.2	14.1
Father	16	11.9	11.9	25.9
Mother	9	6.7	6.7	32.6
Uncle	9	6.7	6.7	39.3
Brother	27	20.0	20.0	59.3
Sister	8	5.9	5.9	65.2
Self	26	19.3	19.3	84.4
Not indicated	19	14.1	14.1	98.5
Many people	1	0.7	0.7	99.3
Cousin	1	0.7	0.7	100.0
Total	135	100.0	100.0	

## 5 Qualitative analysis/respondent theme

This section reports the responses elicited from the students to the open questions posed by the researcher in the questionnaire. Spaces were created in the questionnaire to enable the students provide adequate information on their activities on the social media.

### 5.1 Research question 6

#### 5.1.1 How do secondary students use social media?

Findings from the study revealed the construction of distinct identities by the students on Face book. The students opened their social media accounts using nick names such as: KKhana 7, Kahn Opy lemmy 244, BENJaBenja305, Queen NaaTasha. This shows that the social media is mostly used by students to create an identity or portray themselves in the way they want their peers to see them. Findings from the study further revealed the tendency for bullying and the use of abusive language on social media. This is reflected in the following responses:

*I have opened 2g0 account before, but it was stolen by someone, and the account is Opzy lemmy. So I now opened another it is Opzy lemmy 244. So I now write it on my status that you that stole my account it will not be better for you (Opzy lemmy 244, male, 3 hours daily on social media, single parentage, S1).*

Another student as says:

*It happens when he cursed me and I was angry and started cursing the person till he got angry that he went off line (Questionnaire interview, Male, 13 years old, 30 minutes daily on Facebook, S2).*

These responses above portray a tendency of bullying and the use of abusive language by students on social media. Furthermore, the findings showed the tendency for engaging in pornography by the students. Most of the students interviewed admitted to watching sex movies and engaging in pornography. The names of the sex movies they watch on social media include XXX films, Spartacus, Shoes of Grey, Naked weapon, Alexandral on veranda, White Fuke.

Overall, students' activities on social media range from identity construction, the use of abusive language, bullying and pornography. Also, the study revealed the existence of parental and teacher influence on the activities of the students on social media as many of the students interviewed indicated that their parents and teachers advised them or showed concern about their activities on these platforms. However, this may not have had much effect judging from their activities on social media platforms.

## 6 Discussion and conclusion

### 6.1 Quantitative findings

This discourse has shown that Facebook is the most widely used mobile social network among the selected secondary school students. The ‘World Wide Web’ is replete with literature or accounts of the evolution of the Facebook social networking website. It was created by Zuckerberg to facilitate communication among the Harvard academic community in 2004 (Phillips, 2007). However, what attracted the selected students to Facebook was not explored in this study. Could it be because of the large number of active daily users? Are these students drawn by the verbal and non-verbal features that Facebook afford them? Answers to these questions could be the focus of another study. The emergence of Facebook as the most used social networking website in this study confirms the assertions of previous scholars. Horzum and Demirhan (2017) submitted that Facebook, with 1.23 billion active daily users in 2016, has grown to be one of the preferred social networking websites. Ellison, Steinfield, and Lampe (2007) equally noted that most Facebook users are young people who deployed the platform to overcome the psychological downturn resulting from the loss of connections to old friends who moved to educational colleges. From the observed outcome of this research, it is likely that the opportunity provided by Facebook to users to share information and keep close to their peers after school hours might be responsible for widespread acceptance of Facebook among the respondents.

Ledbetter and Mazer (2013:807) supported the claim that the maintenance of interpersonal ties remains the heartbeat of the core user purpose of the Facebook social networking site. Barbovschi, Machackova, and Olafsson (2015) further explained that social networking websites are used by students to sustain relationships, and that these websites are further used to enlarge social circles and seek new contacts. In the current study, most of the school children that signed up and were operating social media accounts were twelve years old. This age grade is inappropriate to sign up for any social media account. The information provided by the office of the children's e-safety commissioner shows that a child must have attained the age of thirteen to sign up on most social networking websites (https://www.esafety.gov.au/education-resources), and some of the social media service providers, like Vimeo, Yellow, Tinder, Skype, Kik, Keek, and EA (Electronic Arts), demand parental permission from applicants between the ages of 13 and 17 to sign up. The findings show that none of the students signed up on ‘CLUB Penguin’, which is created for 6–14 y old, and ‘Minecraft’, which is appropriate for all age groups, provided minors can document parental permission. A comparison of age and year the respondents signed up on different social media platforms revealed that a large proportion of the school children selected for the study were active on social media sites two to three years before they turned thirteen.

A report by the Daily Mail (November 2014) confirmed the underage use of social media observed in this study. It was reported in the Newspaper that most children had signed up on an online social network by the age of ten. Some of these children between the ages of 8 and 16 years old covertly confessed that they had disregarded the proper age limit for operating a Facebook account. A related report by Aiken (August 2016) on ‘The kids who lie about their age to join Facebook’ revealed that twenty million minors signed up and use Facebook. Aiken (2016) provided further information that these underage users created fake profiles, sometimes with tacit approval from parents. What can educational researchers do to arrest the use of social media by underage school children? It is unlikely that current educational solutions can be used to arrest this trend in the long run. This position is premised on the declaration of Mark Zuckerberg that children should be empowered to use Facebook. Warman (2011) reported that Zuckerberg stated that the educational benefits of signing up on Facebook are so enormous and as a result, children should not be prohibited from using Facebook. Despite the surging incidences of bullying and grooming on Facebook and other popular social networking sites, Warman (May, 2011) reported that Zuckerberg stated that laws prohibiting children below the age of thirteen would be challenged ‘at some point’. In 2011, a report suggested that there were about 7.5 million children below the age of 13 operating accounts on Facebook despite restrictions on younger children in Facebook's terms of service (Spies Shapiro and Margolin, 2014). The data reported by these scholars were collected in 2011, and it is certain that there is an astronomical increase in the number of underage users signing up on social networking sites and using such accounts to socially connect with other people on the social media.

This current study complements previous studies (Sana, 2012; Spies Shapiro and Margolin, 2014; Chassiakos et al., 2016) on the amount of time spent by children on the social media. 87.9% of the children who participated in this study spent a minute to three hours daily on social media. This finding relates to the observation of Spies Shapiro and Margolin (2014) that adolescents and emerging adults spend eleven hours daily on social media, with many of them starting and ending the day checking posts on social networking sites. How does the time spent on the social networking websites affect students learning outcomes? Sana (2012) suggested that students' presence and cognitive absorption on Facebook does not immediately affect their academic achievement. Despite the assertion of this scholar, it is important to ask what happens to the academic achievement of students entangled in social media sites. The results of this analysis showed that majority of the school children selected for the study were assisted by their brothers to open accounts on social networking sites, while others followed the online instructions to sign up. This finding is contrary to the discovery of Boyd et al. (2011) that parents helped their children to sign up on the Facebook despite having awareness of the age limit stipulated in Facebook's terms of service. Contrary to the findings of Boyd et al. (2011), this study showed that the trend among school children on social media is that they either consulted biological brothers to help them sign up on the preferred social media site or signed up by trial and error.

Furthermore, it is observed, from the responses of the selected school children that they learnt to navigate social networking sites through assistance from their biological brothers. The school children selected for this study claimed they learnt to navigate on social networking sites by themselves or were trained to use the sites by their fathers. Unfortunately, the study did not evaluate whether the fathers of the selected school children were aware of the terms of use stipulated by Facebook for users.

### 6.2 Qualitative findings

The analysis of the qualitative data collected from the respondents revealed themes such as cyber pornography, sexting, cyber stalking, abusive language, cyber bullying, and cyber hacking as the major behavioural features of school children on the social media.

#### 6.2.1 Cyber pornography

Cyber pornography is a trend that emerged with the evolution of the Internet. Coetzee (2013) commented that learners are increasingly engaged in the production and distribution of child pornography without realizing the dangers inherent in such a venture. It was evident from the responses elicited from the students that most disagreements they had with other users emerged from the circulation of sexually provocative contents. The emergence of cyber pornography in children's use of social networking sites in this study confirmed Wolak, Finkelhor, and Mitchell's (2012) position that the growing capacity of computers to transmit and store images has the potential to expand the reach of cyber pornography into new populations. These scholars were concerned that such a situation might give rise to child sexual abuse.

#### 6.2.2 Sexting

‘Sexting’ is an emerging term that describes the broadcasting of sexual images by children using messages of text type. Ringrose, Harvey, Gill, and Livingstone (2013) described ‘sexting’ as a ‘portmanteau’ term that bridges the words ‘sex’ and ‘texting’, which connotes the creation, sharing, and distribution of sexually suggestive nude images through mobile phones and the Internet. ‘Sexting’ emerged as another trend among children using social media in this study.

The comment of a respondent (female, Hana 71) that she abused people that sent her a romantic picture on the social media confirmed the findings of Wolfe et al. (2016:634) that sexting is a perilous teenage behaviour that has increased in recent times. Wolfe et al. (2016) cited Lenhart (2009)'s work that reported a teenage girl stating that ‘sexting happens a lot, that her friends did it regularly, and it is not a big deal’ to show the impunity with which students engaged in sexting. The qualitative data showed that girls are more at risk of receiving nude pictures via social media, which confirms the fears of Albury and Byron (2014:139) that teenage girls are at risk of sexting (i.e. vulnerable). Wolfe et al. (2016:636) recommended that schools and homes should monitor the texting and speech activities on schoolchildren's mobile phones to curb sexting-related activities.

#### 6.2.3 Cyber stalking

Another thread that emerged from the respondents' use of social media is cyber stalking. Below is an instance of this:

*It happens when he cursed me and I was angry and started cursing the person till he got angry that he went off line (S2).*

Several scholars (Southworth et al., 2007; D'Ovidio and Doyle, 2003; Fisher et al., 2000) concluded that cyber stalking connotes a variety of behaviours relating to incessant threats and/or molestations, using electronic mails or e-media-based communication that makes a person feel unsafe online and in physical environments. The dimension of stalking among the schoolchildren used for this study closely resembles the phenomenon tagged ‘stalking with technology’ by Southworth et al. (2007). Nobles and Fox (2013) while assessing stalking behaviours described it as a sequence of unwanted, regular, intimidating, or threatening behaviours. The emergence of stalking as an interesting feature of schoolchildren's social media use in this study emphasized the discovery of Lowenstein (2000:161) that stalkers may seek a relationship or be inclined towards aggressive and venereal acts with the victim. In this regard, Wyke (2007) stated that harassment is incessant with unwanted attention, contact, or close observation that trigger fear in the victim.

#### 6.2.4 Abusive language

In discussing the severity of abusive language in the cyberspace, this quotation from a female respondent confirms Andra and Meglich's (2013) and Citron's (2009) assertion that women rarely report cyber gender harassment because people may undermine such a claim or overlook it as ‘just a prank’. Additionally, this foregoing quotation describes another aspect of online abuse which has a deeply gendered structuring (Udupa, 2018). It was evident from this narrative that this abusive language occurrence happened between a male and a female user in the cyberspace. The hurl of curses which is abusive in nature caused the retaliation from this female user of the cyberspace. Andra and Meglich (2013) stated that women are exposed to demeaning and threatening behaviours that made them vulnerable when online and this could reduce their intent to socialise and connect in the cyberspace if personalised coping mechanisms are not adopted. It is expedient to affirm that this narrative confirms that female users of the cyberspace are at risk of being abused than male users. Despite the risks confronting female users in the cyberspace, Udupa (2018) discovered that abuses offer potential opportunities for cyberspace users to participate in online debates of a political nature, and could offer online users to develop the skills to hurl, dodge, or constructively criticise abuses to remain relevant within cyber discursive platforms. The position of Udupa (2018) projects a social importance of abusive language if used as stinging satires to condemn social vices, international discrimination, racism, cyber lawlessness, and other topical social issues.

#### 6.2.5 Cyber bullying

The use of the electronic media to harass other people has been engaged by previous scholars. Cyber bullying could be direct or indirect (See Betts, Gkimitzoudis, Spenser & Baguley, 2017). Betts et al. (2017:1083) posited that cyber bullying of a direct type could be physical, verbal (e.g. utilizing technology to threaten), nonverbal (e.g. sending obscene images), and social (i.e. ostracizing someone from a group while indirect cyber bullying entails spreading gossip, and casting votes on defamatory websites. Dwyer and Easteal (2013:92) in their study on cyber bullying in Australian schools asserted that cyber bullying is a relatively new communication activity that is harmful, exploiting, spiteful, and deleterious to school children. Dwyer and Easteal (2013:92) stated that forms of bullying in cyberspace included harassment, predation, impersonation, sexting, threats, stalking, and any hectoring behaviour through cyber technology.

The children exhibiting these behavioural dispositions are still in the state described by Dwyer and Easteal (2013) ‘as a fait accompli, or something of a rite of passage for children that have not always been given attention by parents and schools. The findings of the current study are consistent with the assertions of Hemphill et al. (2015:2571) that bullying in schools is a social relationship issue. Their study suggested that most students who were victims of cyber bullying were not also victims of traditional bullying, except for twelve percent of students who admitted that they were victims of both cyber and physical space bullying.

#### 6.2.6 Cyber hacking

It is expedient to revisit this finding which suggested cyber hacking for further analysis:

*I have opened 2g0 account before, but it was stolen by someone, and the account is Opzy lemmy. So I now opened another it is Opzy lemmy 244. So I now write it on my status that you that stole my account it will not be better for you (*S1*).*

Internet Safety Campaign Africa (2017) stated that ‘hacking’ which differs from ‘cracking’ is the use of unusually clever and multiplex methods of using computers to gain unauthorized access. The act reported in this statement implies that an unknown person cleverly used a computer connected to the Internet to take over the 2g0 account operated by this respondent. The development of the Internet and other ancillary devices has led to developments in diverse facets of global economy (Ige and Hlalele, 2017). Despite these developments, the Internet and other ancillary devices are susceptible to cyber attacks and other forms of attacks such as cyber hacking reported by this respondent (Holt et al., 2017). Sela-Shayovitz (2012) highlighted the contribution of the media to social construction through the high publicity accorded selected cases of youths that engage in hacking in the popular cultural settings. The incident of cyber hacking reported by this respondent affirmed Sela-Shayovitz's (2012) assertion that cyber hackers mainly conduct their activities via the Internet which acts as a veritable platform for the formation and sustenance of hacking activities that are conducted using chat rooms and bulletin boards. It was evident from this quotation that the 2go account was taken over by an unknown person who had a superior knowledge of how the Internet works. An interesting point that emerged from this quotation is that modern communication resources such as the Internet, mobile phones, and common computing devices could be compromised through diverse forms of cloud attacks such as cyber hacking, and distribution of malicious softwares (Holt et al. 2017).

## 7 Implications for civics and social studies teachers

The findings of the current study have promising implications for teachers regarding civics or ethics education. This study has shown that communication among students in 21^st^ century schools goes beyond their physical interaction in school compounds to include online as well. These findings accentuate why teachers and parents should pay attention to students' activities on the social media. The outcome of this study has thrown up questions that require intellectual discourse among teachers to answer.

Who controls the activities of school children on social media? Teachers acting *in loco parentis* or parents? Do scholars agree that this role falls within the ambit of school teachers? Is this role captured by the teaching regulations across transnational boundaries? If not, how do schools handle these ethical scenarios when schoolchildren engage in activities that conflict with school norms and values on the social media? If another school of thought feels it is the duty of the parents, do the myriads of professional commitments permit parents to effectively monitor the activities of schoolchildren on the social media? Where the parents have a lighter professional commitment, are they aware that their children or wards have signed up for a social media account? If not, how will such parents manage a situation they are not aware of?

These questions might be the focus of future studies examining the ethical dimensions relating to teachers' moderation of students' activities on the social media. In the light of the findings of this study, it is recommended that schools infuse the relevant aspect of the Civics/Social Studies curriculum with concepts that are of direct relevance to children's social media use. Considering the moral and legal implications of teachers arbitrating on disagreements among students on the social media, teachers are advised to adopt educative techniques to resolve such conflicts among students. Burke and Kraut (2016) noted that despite consensus that the connection between social networking sites and students' well-being is dependent on how these technologies are used, scholars agree less to a lesser extent on what constitutes the factors integral to this usage. One of these integral factors which has received little attention is the ethical dimension of children's use of the social media in global schools.

There are growing questions on the impact of new communication technologies on children's psycho-social well-being (See Burke and Kraut, 2016; Burke et al., 2011). The impact of obnoxious posts by friends and anonymous people on social networking sites demands urgent referrals of such emotionally traumatized school children to school counsellors. However, further research might be appropriate to evaluate how well counsellors in developing nations are equipped to help children deal with this new locus of social engagement.

It was evident from the response of many of the selected students that most of the constructive guidance they received came from volunteers of the National Youth Service Scheme (N.Y.S.C) in Nigeria. These volunteers are graduates from higher institutions of learning in Nigeria. The Scheme was set up on 22 May 1973 to involve graduates of Nigerian descent from higher institutions of learning in Nigeria and other parts of the world in community service that meets the developmental needs of the nation. It is recommended that schools entrust these youth corps members with the task of orientating school children on the appropriate use of the social media in Nigeria.

## 8 Limitations and future studies

Researchers should be circumspect in transferring or generalizing the findings of this study. The study has a small sample that was randomly selected from Southern Nigeria. This sample of schoolchildren was selected from urban schools in the first year of schooling at the junior secondary school level in Nigeria, a developing nation. Researchers should exercise severe caution in generalizing the findings of this study to school children in developing nations.

The qualitative interview guide used to collect the data in this study was developed in collaboration with some teachers in the schools selected for the study, who did not participate in the study. Research has shown that it might not be possible to separate the respondents' representations from the effect of the interview context (Doise, 1995; Thornberg, 2010). Despite these limitations, this study has provided a point of departure for other studies to investigate the ethical aspect of children's social media use. The findings have also provided information on the activities of schoolchildren on social networking sites (SNS), and the demographical information used to operate such SNS accounts. It is hoped that the findings of this study will stimulate further studies on the use of social media by school children.

## Declarations

### Author contribution statement

Olugbenga A Ige: Conceived and designed the experiments; Performed the experiments; Analyzed and interpreted the data; Contributed reagents, materials, analysis tools or data; Wrote the paper.

### Funding statement

This research did not receive any specific grant from funding agencies in the public, commercial, or not-for-profit sectors.

### Competing interest statement

The author declares no conflict of interest.

### Additional information

No additional information is available for this paper.
