# Supplementary material for: What we do on social media! Social representations of schoolchildren's activities on electronic communication platforms
Source: Heliyon. 2020 Aug 20;6(8):e04584. doi: 10.1016/j.heliyon.2020.e04584 (PMC7452393; doi:10.1016/j.heliyon.2020.e04584)
Supplement: Supplementary file 1 — Appendix [file mmc1.pdf]

Appendix

SOCIAL NETWORKING MEDIA USAGE QUESTIONNAIRE (SNMUQ)

STUDENT'S QUESTIONNAIRE

Dear Student,

This research instrument aims to evaluate the use of social networking websites use among school children. Kindly respond to this questionnaire as honest as possible.

Your signature and Date (If you agree to participate in this study):_____________________

SCHOOL NAME

____

STUDENT NUMBER (9 digits):

CLASS (e.g. 3A)

____

GENDER (✓) Boy ( ) Girl ( ) **DATE OF BIRTH:** **Age:** **Tribe:**

Home Background

I live with my father and mother ( ) I live with my father only ( ) I live with my mother only ( )

I live with my Grandmother ( ) I live with my Aunty ( ) I live with my Grandfather ( )

1. What social media websites do you use?

1. Facebook ( ) 2. LinkedInn ( ) 3. Twitter ( ) 4. 2go ( ) 5. Youtube ( ) 6. Any other not listed:_____________________

2. How many minutes/hours do you spend on the selected social media sites each day____________?

3. Do you have a mobile phone?________ If Yes, which line and network(MTN, GLO, AIRTEL etc) ____

4. Do you connect to connect to the selected social media with your phone? Yes ( ) No ( )

5. Is your parent/guardian aware you connect to social media via your phone?

6. What name did you use to open your facebook/2go account?_____________________

7. In which year did you open facebook/2go account?________________________

8. Who assisted you to open facebook/2go account?_________________________

9. Have you abuse anyone that anyone that offended you before using the facebook/2go? Yes ( ) No ( )

If yes, how did it happen?____

____

____

10. Do watch 'mojo' i.e. sex films on your mobile phone when connected to the Internet? Yes ( ) No

If yes, mention titles of the
films____

11. Has your at parents anytime restricted your use of social media usage?______________________________
____
____
____
____
____

12. Which of your teachers have counsel you on the use of social media in the past?_______________________
____
____
____
____
____

13. If someone does not respond to your friendship request on the facebook/2go, what will you do to him/her?
____
____
____
____
____
____
____

14. Who taught you to use facebook/2go?____
